# Application of a library of near isogenic lines to understand context dependent expression of QTL for grain yield and adaptive traits in bread wheat

**DOI:** 10.1186/s12870-016-0849-6

**Published:** 2016-07-19

**Authors:** Alba Farré, Liz Sayers, Michelle Leverington-Waite, Richard Goram, Simon Orford, Luzie Wingen, Cathy Mumford, Simon Griffiths

**Affiliations:** Department of Crop Genetics, John Innes Centre, Norwich, NR4 7UH UK

**Keywords:** Yield, Wheat, Near isogenic lines, QTL

## Abstract

**Background:**

Previous quantitative trait loci (QTLs) studies using the Avalon × Cadenza doubled haploid (DH) population identified eleven QTLs determining plant height, heading date and grain yield. The objectives of this study were: (i) to provide insight into the effects of these QTLs using reciprocal multiple near isogenic lines (NILs) with each pair of alleles compared in both parental backgrounds (Avalon or Cadenza), (ii) quantifying epistasis by looking at the background effects and (iii) predict favourable allelic combinations to develop superior genotypes adapted to a target environment.

**Results:**

To this aim, a library of 553 BC_2_ NILs and their recurrent parents were tested over two growing seasons (2012/2013 and 2013/2014). The results obtained in the present study validated the plant height, heading date and grain yield QTLs previously identified. Epistatic interactions were detected for the 6B QTL for plant height and heading date, 3A QTL for heading date and grain yield and 2A QTL for grain yield.

**Conclusion:**

The marker assisted backcrossing strategy used provided an efficient method of resolving QTL for key agronomic traits in wheat as Mendelian factors determining possible epistatic interactions. The study shows that these QTLs are amenable to marker assisted selection, fine mapping, future positional cloning, and physiological trait dissection.

**Electronic supplementary material:**

The online version of this article (doi:10.1186/s12870-016-0849-6) contains supplementary material, which is available to authorized users.

## Background

Many of the key traits for wheat such as yield, flowering time, and canopy architecture are quantitatively inherited, displaying continuous variation due to the contribution of multiple genes, each with relatively small effects, called quantitative trait loci (QTL). Greater understanding of the physiological and molecular basis of individual QTL is a major goal in the identification of fundamental biological processes controlling them and guiding improved manipulation of higher level quantitative traits. One way to achieve this is to convert quantitative to qualitative traits through the production of Near Isogenic Lines (NILs). For bread wheat QTLs this has been achieved in just a few instances eg [1, 2]. This study represents a process of systematic QTL validation by the production of NILs in a multi stream backcrossing strategy that not only serves as a validation platform for the majority of QTLs identified in Avalon × Cadenza UK reference segregating population, but also as a unique tool for the further understanding of context dependent QTL expression.

The major target in wheat breeding is grain yield (GRYLD). Increasing GRYLD is challenging for breeders due to its complexity, environmental and genetic interactions and low heritability. To reduce the complexity of GRYLD it can be broken down into numerical components: grain number (GRpsqm) and thousand grain weight (TGRWT) which are often negatively associated [3, 4]. GRpsqm is the strongest historical determinant of GRYLD increases (usually due to the number of grains per spike) [4–6] and currently the yield component used to improve GRYLD potential in wheat breeding [7]. GRpsqm is also one of the most plastic [8] and least heritable [9] of the yield components, so precise genetic stocks, which allow the precise quantification of genetic and environmental interactions are essential for deeper genetic analysis of this trait. Potential improvement in GRYLD driven by GRpsqm can only be fully realised if increases in GRpsqm are complemented with improvements in TGRWT, while avoiding compensatory effects (trade-off) between both yield components [10, 11]. TGRWT can be further dissected into individual components including grain length (GRL), width and area. GRL was found to be positively associated with TGRWT [10]. The identification of QTLs in the same position for TGRWT and GRL support the idea that GRL is a key trait for TGRWT and it is possible to achieve higher yields with large grains [11]. Several studies have identified QTLs for GRYLD and grain yield–related traits [12–28].

Lying beneath the numerical components of grain yield are a complex web of developmental traits, the genetic control of which has just as an important influence on the final expression of GRYLD, as well as crop adaptation, and must be considered in wheat breeding. These include crop height (PH) and heading date (HD) which are more stably inherited. Short stature and optimal flowering are breeding pre-requisites for achieving high yield varieties. Reduced PH has been exploited by the introduction of *Rht* dwarfing genes (*Rht-B1*b, *Rht-D1*b) which reduce lodging, increase harvest index and yield potential [29, 30]. *Rht-B1* (on 4BS) and *Rht-D1* (on 4DS) reduce PH by reducing the response to gibberellin with pleiotropic effects on grain number and yield [31]. The increase in grain yield and grain number was driven by the increase in grains per spike [29, 30, 32]. Another dwarfing gene, *Rht8*, on 2DS has also been used to reduce stature and improve lodging resistance, without yield penalty [33, 34]. Gasperini et al. [33] determined that the reduced stature is a consequence of shorter internodes with the largest differences obtained in the peduncle and the first internode. In addition to the major crop height genes, other QTLs for PH have been identified in a number of studies [35].

Wheat is the most widely grown grain crop in the world because of its broad geographical adaptation. Part of this adaptation is due to the manipulation of genes that control HD (Photoperiod (*Ppd*), Vernalization (*Vrn*) and ‘*Earliness per se*’ (*Eps*) genes). *Ppd* genes control day length response, *Vrn* genes determine the requirement for a period of cold, *Eps* genes are important for the fine-tuning of flowering time and account for the variation in HD after photoperiod and vernalization requirements are fully satisfied. QTLs for HD and PH have been found in the same position, with early alleles associated with increased height in the Avalon × Cadenza DH population [36, 37]; this could therefore, be a trade-off regarding yield manipulation. QTLs for HD in wheat have been identified in several studies [13, 38–43].

Using the Avalon × Cadenza DH population as a platform, Ma et al. [37] suggested that the ideal UK target genotype should be: PH and HD similar to Avalon; *Rht-D1b* and *Vrn-A1b* alleles; high GRpsqm and TGRWT; long and wide grains and maximum GRYLD. Breeding for superior genotypes can be achieved through the combination of favorable alleles using marker-assisted selection (MAS). To this aim, NILs have to be developed to validate the QTLs identified in the Avalon × Cadenza DH population for PH [36], HD [38] and GRYLD [37]. The objectives of this study were: (i) to validate these QTLs using the NILs developed by MAS using two recurrent parents (Avalon and Cadenza), (ii) determine possible epistatic interactions and (iii) define the expected genotype if the target regions were introgressed in the same background in order to to develop superior genotypes adapted to a target environment.

## Methods

### Plant material and genotyping

The Avalon × Cadenza DH population was developed as a tool for the genetic discovering of wheat, as part of the Wheat Genetic Improvement Network (WGIN) (www.wgin.org.uk) and represents a broad spectrum of elite UK winter germplasm produced in different UK wheat breeding programmes. The parents were chosen for contrasting canopy architecture traits. Avalon and Cadenza carry recessive photoperiod sensitive alleles of *Ppd-D1* and *Ppd-B1*. Avalon (UK winter wheat) carries recessive alleles of *Vrn-A1*, *Vrn-B1* and *Vrn-D1* whereas Cadenza (UK alternative wheat) carries the dominant *Vrn-A1a* allele. The parents also differ for *Rht-D1*, Avalon carries the dwarf allele *Rht-D1b* whereas Cadenza carries the wild type allele (*Rht-D1a*).

**Fig. 1:**
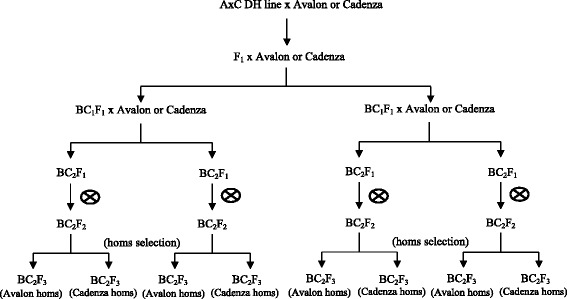
NIL development scheme (homs = homozygotes)

QTLs controlling flowering time, plant height and grain size and shape had been previously identified in the Avalon × Cadenza DH population using meta-QTL analysis [15, 36, 38]. Eleven QTLs, on chromosomes 1B, 1D, 2A, 2D, 3A, 3B, 5A, 6A, 6B, 7B and 7D were chosen as target regions for introgression in our marker assisted backcrossing scheme (Fig. 1). A total of 31 microsatellite (SSR) markers linked to the target QTLs were used for foreground selection (Table 1).

**Table 1:** SSR markers used for NILs development

QTL region	Trait	Marker	Reference
1B	HD	wmc44 – barc80	[1, 3] HD ↓ PH ↑
1D	HD	gdm111	[1, 3, 4] HD – GRYLD – L/W ↑ TGRWT - GRW↓
2A	PH	gwm359 – gwm122	[2, 3] PH – GRYLD - TGRWT – GRL ↑
2D	PH	cdf36 – gwm261	[2] PH ↓
2D	GRYLD	wmc18 – gwm539	[3, 4] GRYLD ↓ GRL – L/W ↑
3A	PH- GRYLD	gwm369 – wmc505 – barc19 – wmc264	[1–4] HD - PH - GRYLD ↓ GRL – L/W ↑
3B	PH	cfd79b – gwm285 – wmc326	[2, 3] HD ↓ PH ↑
3B	GRYLD	gwm389 – barc75 – gwm493	[3, 4] GRYLD – TGRWT - GRA – GRL – GRW ↑
5A	GRYLD	gwm156a – gwm186	[3, 4] TGRWT - GRA – GRL – L/W ↓
6A	PH	barc23a – barc171 – gwm570	[1, 2] HD ↓ PH ↑
6B	PH	wmc105 – gwm219	[1, 2] HD - PH ↓
7B	GRYLD	barc176 – wmc517 – gwm577	[1, 3] HD ↓ PH ↑
7D	GRYLD	cdf21a – psp3113	[3, 4] PH - GRL – L/W ↓

(HD = Heading date, PH = Plant height and GRYLD = Grain yield. On 3A, HD and PH QTLs collocate in the same region in the original Avalon × Cadenza DH population. [1] Griffiths et al. 2009, [2] Griffiths et al. 2012, [3] Ma et al. 2015; [4] Gegas et al. 2010; ↑ and ↓ indicated that the Avalon allele increased or reduced, respectively, in the original QTL study)

Donor Avalon × Cadenza DH lines were crossed with recurrent parents (either Avalon or Cadenza) to produce F_1_ plants. These plants were grown and backcrossed twice with the recurrent parent to produce two independent BC_2_ streams. After each backcross, progenies were genotyped at the QTL of interest, and those heterozygous at the QTL were carried forward to the next cross. BC_2_ individuals were then self fertilised and homozygous (carrying Avalon or Cadenza alleles in the QTL region) were selected with flanking SSR markers, self fertilised and provided seeds for multiplication and then large scale experiments. The BC_2_ derived NILs contain an estimated recurrent parent genomic background of at least 93.75 %. A total of 553 BC_2_ NILs were generated (250 and 303 NILs with Avalon and Cadenza background, respectively; Table 2). More details of the development of the Avalon × Cadenza NIL population and streams pedigree are available in WGIN website (www.wgin.org.uk).

**Table 2:** Number of NILs used for each chromosome based on the introgression allele and background

Background	Allele	QTL region										
		1B	1D	2A	2D	3A	3B	5A	6A	6B	7B	7D
Avalon	Avalon	3	8	9	37	14	4	8	22	26	4	4
	Cadenza	7	5	2	37	12	6	8	8	20	2	4
Cadenza	Avalon	8	19	8	27	28	13	-	26	15	-	-
	Cadenza	9	22	5	33	23	17	-	22	26	-	-

### Experimental design and phenotyping

Field trials were conducted at Church Farm, Bawburgh, Norfolk (UK) in two growing seasons (2012/2013 and 2013/2014). Sowing date differed between trials, the 2012–2013 trial was sown the first week of November 2012 whereas the second trial was sown the second week of October 2013. Church Farm has a sandy clay loam soil type. Fungicides and fertiliser products were applied according to standard agronomic practice. The experimental design for both trials consisted of a randomized complete block design with three replicates (some NILs had more replicates) and the two parents (Avalon and Cadenza). The experiment contained a total of 1800 and 1868 plots in 2012/2013 and 2013/2014, respectively. The additional plots in 2013/2014 were due to an increase number of replicates for the parents. Each plot consisted of 7 rows 20 cm apart and 4 m long. A sowing rate of 260 seeds.m^−2^ was used in all cases. An additional file shows the details of experimental conditions in more detail (see Additional file 1). Heading date was scored as the day when 50 % of the ears in a plot were more than 50 % emerged from the flag leaf and was assessed in thermal time (HD) (°C days, using a base temperature of 0 °C). Plant Height (PH) was measured from soil level to the top of each wheat ear. Grain Yield (GRYLD) was recorded per plot in both years. Prior to harvest, 10 main shoots were sampled from each plot for assessments of yield components including thousand grain weight (TGRWT), grain width (GRW), grain length (GRL) and grain area (GRA) using a Marvin digital seed analyser (GTA Sensorik GmbH). Grain number (GRpsqm) was estimated from GRYLD and TGRWT. PH components (ear length (EL), peduncle (PL) and internode lengths (from the first to the fifth counted from the top, abbreviated as 1stITL, 2ndITL, 3rdITL, 4thITL, 5thITL)) and GRpsqm components (spikes/m^2^ (S), spikelet/spike (s/S) and grains/spikelet (G/S)) were only measured in 2013. Harvest Index (HI) and Biomass was only measured on 3A NILs. This was done by selecting an internal row from the plot and selecting a 60 cm section of this row that was judged to be representative of the whole plot. Plants within this strip were then cut to ground level and threshed so that the ration of grain yield to total above ground biomass could be calculated.

### Statistical analyses

In order to remove spatial effects and to estimate heritabilities a mixed model was fitted for all traits. The model used included environment, replicates within environment and checks within environment as fixed factors, and row (of plots) within environment, column within environment, genotype (NIL lines) and the interaction between genotype x environment as the random factors. Narrow-sense heritabilities were estimated using the variance components from this model, as follows: h^2^ = σ^2^_g_ / [σ^2^_g_ + (σ^2^_ge_/e) + (σ^2^_res_/re)]; where σ^2^_g_ is the variance component of genotype; σ^2^_ge_ is the variance component of the interaction genotype x environment; σ^2^_res_ the error; e, the number of environments, and r, the number of replicates per trial.

Best Linear Unbiased Estimators (BLUEs) were estimated for each genotype within individual trials considering genotype as fixed, and replicate, row and column as random factors. The estimated BLUEs were used to determine significant difference between Avalon and Cadenza alleles in the target QTL regions using linear mixed models. The first model included as fixed factors the environment, background, allele nested within QTL and the 2, 3 and 4-level interactions, while the random factor was the remaining genotypic variance. All analysis were conducted using Genstat 16^th^ [44].

## Results

Eleven QTLs for three agronomic traits (HD, PH and GRYLD) in the two genetic backgrounds of the bi-parental Avalon × Cadenza DH population, which were used for their discovery, were investigated using 553 BC_2_ NILs and their recurrent parents in the field for two years. The mean values of the two parents (Avalon and Cadenza) and the NILs in each environment are shown in Table 3. In all cases, Cadenza was taller and flowered earlier than Avalon (with a difference of 3.14 cm and 14 °C days to heading in 2013 and 6.12 cm and 24.90° days to heading in 2014). In 2013 Avalon and Cadenza had similar grain yield (8.27 T/Ha and 8.11 T/Ha, respectively). However, in 2014, Avalon had lower grain yield (10.45 T/Ha) than Cadenza (11.56 T/Ha). These differences can be explained dissecting GRYLD into its components (GRpsqm and TGRWT). Avalon grown in 2013 had greater GRpsqm and TGRWT compared to 2014. The mean values of GRA and grain shape (L/W) for the parents were consistent in both years; Avalon had lower GRA and higher L/W ratio than Cadenza. There were no significant differences between the parental lines for most of the traits with the exception of PH in 2013. However, in 2014, all the traits except GRA (*P* = 0.301) and GRpsqm (*P* = 0.004) showed significant differences at *P* < 0.001 (Table 3).

**Table 3:** Mean phenotypic values of the two parents and 553 BC_2_ NILs for each environment

Year	Trait	Parents		NILs				
		Avalon	Cadenza	Mean	Range	Avalon background mean	Cadenza background mean	H^2^
2013	HD	1358.00 (8.00)	1344.00 (7.19)	1353.71 (1.33)	1287 - 1430	**1368.84**	**1341.22**	0.82
	PH	**69.50 (0.62)**	**72.64 (0.62)**	71.64 (0.10)	58.65 - 82.35	**70.98**	**72.20**	0.81
	GRYLD	8.27 (0.18)	8.11 (0.22)	7.92 (0.03)	5.01 - 9.70	7.89	7.95	0.38
	TGRWT	48.32 (0.45)	47.22 (0.60)	46.93 (0.09)	35.64 - 56.22	46.73	47.09	0.59
	GRpsqm (x10^3^)	17.14 (335.58)	17.04 (408.05)	16.87 (56.73)	11.27 – 22.40	16.86	16.88	0.48
	GRA	21.22 (0.23)	21.23 (0.21)	20.32 (0.03)	16.58 - 23.67	20.26	20.37	0.19
	GRL	6.92 (0.03)	6.92 (0.02)	6.79 (0.00)	6.21 - 7.18	6.79	6.79	0.49
	GRW	3.83 (0.03)	3.85 (0.03)	3.73 (0.00)	3.28 - 4.18	3.71	3.75	0.16
	L/W	1.81 (0.01)	1.80 (0.01)	1.82 (0.00)	1.65 - 2.02	1.83	1.82	0.38
2014	HD	**1775.71 (1.68)**	**1750.81 (2.11)**	1768.53 (0.56)	1717 - 1848	**1782.96**	**1756.63**	
	PH	**80.26 (0.50)**	**86.38 (0.48)**	84.48 (0.16)	70.11 - 106.26	**82.16**	**86.40**	
	GRYLD	**10.45 (0.13)**	**11.56 (0.15)**	10.93 (0.03)	8.20 - 14.13	**10.51**	**11.28**	
	TGRWT	**52.52 (0.29)**	**54.47 (0.35)**	53.46 (0.07)	45.62 - 58.96	**52.49**	**54.26**	
	GRpsqm (x10^3^)	19.98 (319.38)	21.29 (316.38)	20.49 (65.97)	15.63 – 26.61	**20.06**	**20.84**	
	GRA	23.42 (0.11)	23.58 (0.11)	23.43 (0.02)	21.25 - 25.30	**23.25**	**23.57**	
	GRL	**7.25 (0.02)**	**7.17 (0.01)**	7.18 (0.00)	6.82 - 7.46	**7.20**	**7.17**	
	GRW	**4.04 (0.01)**	**4.11 (0.01)**	4.08 (0.00)	3.87 - 4.30	**4.04**	**4.11**	
	L/W	**1.79 (0.00)**	**1.74 (0.00)**	1.76 (0.00)	1.63 - 1.87	**1.78**	**1.74**	

Values highlighted in bold are significantly different at *P <* 0.001. Stardard error of the mean is included in brackets (HD = Heading date (°C), PH = Plant height (cm), GRYLD = Grain yield (T/Ha), TGRWT = Thousand grain weight (g), GRpsqm = Grains per square meter, GRA = Grain area (mm^2^), GRL = Grain length (mm), GRW = Grain width (mm) and L/W = Grain shape)

**Fig. 2:**
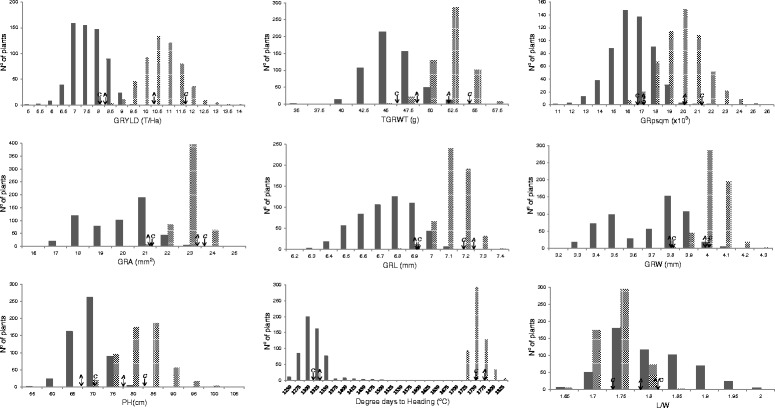
Frequency distribution of NILs BC_2_ lines for GRYLD, TGRWT, GRpsqm, GRA, GRL, GRW, PH, HD and the L/W ratio in 2013 (dark bars) and 2014 (pattern bars). Values for parents are indicated with arrows (A = Avalon; C = Cadenza)

Means of the NILs for GRYLD values ranged from 5.01 to 9.70 and 8.20 to 14.13 T/Ha in 2013 and 2014, respectively (Table 3; Fig. 2). For PH, the values ranged from 58.65 to 82.35 cm in 2013 and 70.11 to 106.26 cm in 2014. For HD, the values ranged from 1287 to 1430 and 1717 to 1848° days to heading in 2013 and 2014, respectively. This large genetic variability reflected transgressive segregation since differences between parents were smaller than the range of NILs lines indicating the polygenic inheritance of the trait alleles. Distributions of NILs lines were approximately normal in all cases with the exception of GRA and GRW in 2013. NILs can be classified in two backgrounds depending on the recurrent parent (Avalon or Cadenza) used in the marker assisted backcrossing scheme. Significant differences of the mean of NILs with the Avalon versus Cadenza background were detected for all the traits in 2014; however, only HD and PH, were significant in 2013 (Table 3). NILs with the Avalon background were shorter, flowered later and had lower grain yield than NILs with the Cadenza background. Overall, the mean values of the NILs were similar compared to the recurrent parent used in each case for all the traits in both years with the exception of GRYLD, GRpsqm and TGRWT in 2013.

These results showed a significant background × year interaction that can be partially explain by the difference between the two growing seasons (2012/2013 and 2013/2014) in terms of temperature and precipitation during the growing cycle (see Additional file 1). To meteorologically characterize each environment, we arbitrarily defined three consecutive ‘phenophases’: vegetative phase (from sowing until March), reproductive phase (from April until flowering) and grain filling phase (one month after flowering). Overall, the rainfall during 2014 (591 mm) was higher than in 2013 (471 mm) throughout the growing cycle. For each phenophase, the biggest difference was observed in the grain filling phase (14.7 and 30.1 mm in 2013 and 2014, respectively), followed by the reproductive phase (98.6 mm in 2013 and 153.4 mm in 2014) and finally the vegetative phase (357.8 mm in 2013 and 407.5 mm in 2014). In terms of temperature, the biggest difference was observed during the vegetative phase (604.15 and 1240.45° days in 2013 and 2014, respectively), followed by the grain filling phase (943.10° days in 2013 and 678.00° days in 2014) and finally the reproductive phase (520.30 and 415.85° days in 2013 and 2014, respectively). The duration of each phenophase is generally determined by interactions between genetical and environmental factors such as temperature and rainfall (these effects differ depending on when the factors act). Increasing temperatures, in general, accelerate phenological development resulting in shorter developmental phases and reduce GRYLD in the absence of terminal heat and drought [45]. Another important factor to consider, which differed between trials, is the sowing date as this can also affect the duration of the phenophases. In our study, the 2012–2013 trial was sown in November 2012 whereas the second trial was sown in October 2013. The combination of early sowing with favourable meteorological conditions (wetter and warmer) resulted in taller plants, earlier flowering and increased GRYLD and yield components in 2014. Our data suggested that GRYLD is largely determined by GRpsqm which is established by anthesis and can be reduced as a consequence of water availability and/or high temperatures during the critical period (20 days before anthesis and 10 days late. The incresased GRpsqm observed in 2014 was therefore partially due to high rainfall and slightly lower temperatures during this phase. Lower night-time temperatures would also reduce rates of respiration making more photosynthetic products available for growth and development. TGRW is determined by the rate and duration of grain filling which is driven by genetic factors (cultivar) and environmental factors (temperature) [46–48]. The temperature during grain filling was slightly lower in 2014 but higher rainfall was observed. In addition, GRpsqm and TGRW can also be positively influenced by the duration of the vegetative phase which has a positive effect on GRYLD.

Correlations among traits were analysed for each environment and summarized in Table 4. Correlation coefficients were low to moderate among GRYLD, PH and HD. There was no significant correlation of HD, with GRYLD, or PH in 2013 (0.07 and −0.04, respectively). A statistically significant correlation was found between PH and GRYLD (0.48). However in 2014, the correlations between traits were 0.25, −0.16 and −0.37 for GRYLD – PH, GRYLD – HD and PH – HD, respectively, indicating that early flowering genotypes were associated with higher grain yield and taller plants. GRYLD was highly positively correlated with GRpsqm (0.80 in 2013 and 0.89 in 2014) and displayed lower positive correlation with TGRWT (0.31 and 0.22 in 2013 and 2014, respectively). As expected, a significant negative correlation was found between TGRWT and GRpsqm (−0.31 in 2013 and −0.24 in 2014). In a high yielding year, like 2014, TGRWT showed high significant correlation with GRA (0.87) and GRW (0.86) and was moderately correlated with GRL (0.38). Measures of grain size (GRA and TGRWT) were not independent of grain shape (L/W) as significant negative correlations were obtained between GRA and L/W (−0.79 in 2013 and −0.33 in 2014) and TGRWT and L/W (−0.36 and −0.54 in 2013 and 2014, respectively). Broad sense heritabilities ranged from 0.16 to 0.82 with PH and HD showing the highest heritability across years (Table 3).

**Table 4:** Genetic correlations between GRYLD, TGRWT, GRpsqm, GRA, GRL, GRW, PH, HD and the L/W ratio in the 553 BC_2_ NILs in 2013 (below the diagonal) and 2014 (above the diagonal)

	GRA	GRpsqm	GRYLD	L/W	GRL	PH	TGRWT	HD	GRW
GRA	**-**	**−0.29**	*0.11*	**−0.33**	**0.67**	**0.38**	**0.87**	**−0.38**	**0.84**
GRpsqm	−0.10	-	**0.89**	−0.06	**−0.31**	0.04	**−0.24**	0.06	**−0.14**
GRYLD	**0.21**	**0.80**	-	**−0.31**	−0.13	**0.25**	**0.22**	**−0.16**	**0.25**
L/W	**−0.79**	0.01	**−0.21**	-	**0.46**	**−0.36**	**−0.54**	**0.38**	**−0.78**
GRL	**0.89**	*−0.13*	**0.14**	**−0.43**	-	0.07	**0.38**	−0.03	**0.19**
PH	*0.12*	**0.27**	**0.48**	−0.08	0.10	-	**0.45**	−**0.37**	**0.45**
TGRWT	**0.51**	**−0.31**	**0.31**	**−0.36**	**0.45**	**0.33**	-	**−0.49**	**0.86**
HD	−0.03	**0.26**	0.07	0.00	−0.05	−0.04	**−0.29**	-	**−0.44**
GRW	**0.97**	−0.07	**0.22**	**−0.91**	**0.76**	0.11	**0.47**	−0.03	-

Italics and bold letters are *P-*values under the null hypothesis that the correlation is statistically different from zero for *P* < 0.01 and *P* < 0.001, respectively

In order to determine if there was a significant difference between Avalon and Cadenza alleles in the target QTL region, based on the background and year, a mixed model was carried out for all the traits. The results are showed in Tables 5 and 6.

**Table 5:** Summary of the mixed model analysis

Source of variation	d.f.	PH (cm)		HD (^o^ C)		GRYLD (T/Ha)		GRpsqm		TGRWT (g)	
		Wald-test	p-value	Wald-test	p-value	Wald-test	p-value	Wald-test	p-value	Wald-test	p-value
Year (Y)	1	2998.52	<0.001	189091.70	<0.001	3954.32	<0.001	1457.72	<0.001	1996.79	<0.001
Background (B)	1	126.57	<0.001	802.89	<0.001	32.24	<0.001	0.29	0.590	72.87	<0.001
Chromosome (C)	10	102.71	<0.001	74.21	<0.001	51.96	<0.001	83.69	<0.001	66.33	<0.001
Y.B	1	40.11	<0.001	0.35	0.552	73.5	<0.001	30.26	<0.001	20.97	<0.001
Y.C	10	28.42	0.002	28.86	0.001	9.75	0.463	16.75	0.082	20.98	<0.001
B.C	7	124.02	<0.001	129.03	<0.001	43.82	<0.001	67.35	<0.001	62.38	<0.001
C(Allele)	11	260.96	<0.001	241.09	<0.001	62.07	<0.001	93.18	<0.001	106.53	<0.001
Y.B.C	7	10.48	0.164	14.63	0.042	7.03	0.220	7.54	0.185	4.45	0.487
Y.C(Allele)	11	14.70	0.199	39.67	<0.001	12.7	0.316	9.70	0.558	11.86	0.376
B.C(Allele)	8	15.59	0.050	22.87	0.004	8.68	0.194	6.38	0.383	3.19	0.785
Y.B.C(Allele)	8	1.26	0.996	5.92	0.657	5.73	0.455	6.80	0.341	5.87	0.439
	Chromosome	PH (cm)		HD (^o^ C)		GRYLD (T/Ha)		GRpsqm		TGRWT (g)	
		Allele		Allele		Allele		Allele		Allele	
		Av.	Cd.	Av.	Cd.	Av.	Cd.	Av.	Cd.	Av.	Cd.
	1B	79.15	79.86	**1552**	**1572**	**9.43**	**9.79**	18955	19415	49.89	50.26
	1D	77.71	76.41	**1574**	**1548**	9.63	9.33	**18929**	**18167**	50.49	51.17
	2A	77.91	77.36	**1567**	**1557**	9.21	9.46	18382	18471	**49.79**	**50.93**
	2D	**77.11**	**81.77**	1560	1554	9.75	9.91	19195	19800	50.45	49.76
	3A	**74.71**	**79.96**	**1555**	**1580**	**9.12**	**9.74**	**18185**	**19515**	49.81	49.72
	3B	**79.13**	**74.58**	1566	1568	9.65	9.94	18604	19117	51.71	51.71
	5A	73.93	75.65	1576	1573	8.92	9.00	17891	16985	**49.52**	**52.82**
	6A	**79.48**	**74.69**	**1558**	**1574**	9.33	9.28	**18088**	**18928**	**51.40**	**48.69**
	6B	75.62	77.24	**1556**	**1570**	9.42	9.63	**18800**	**19465**	49.92	49.21
	7B	75.42	73.72	1592	1582	**9.84**	**9.03**	**19682**	**17812**	49.67	50.18
	7D	**74.11**	**79.40**	1552	1572	9.45	9.30	19072	18920	49.34	49.06
	Average s.e.d	0.869		3.535		0.155		314.2		0.462	

Analysis performed for all the agronomic traits studied comprising 553 NILs carrying the Avalon or Cadenza alleles in the introgressed region in two trials (the significant levels are based on the Wald test). (HD = Heading date, PH = Plant height, GRYLD = Grain yield, TGRWT = Thousand grain weight, GRpsqm = Grains per square meter, GRA = Grain area, GRL = Grain length, GRW = Grain width and L/W = Grain shape)

Bold text indicates that the difference between the means of the NILs carrying the Avalon and Cadenza allele is greater than twice the standard error of the difference

**Table 6:** Summary of the mixed model analysis

Source of variation	d.f.	GRA (mm^2^)		GRL (mm)		GRW (mm)		L/W	
		Wald-test	*p*-value	Wald-test	*p*-value	Wald-test	*p*-value	Wald-test	*p*-value
Year (Y)	1	5019.60	<0.001	2932.81	<0.001	3826.67	<0.001	492.50	<0.001
Background (B)	1	23.38	<0.001	2.11	0.146	110.89	<0.001	139.62	<0.001
Chromosome (C)	10	780.46	<0.001	262.91	<0.001	1204.83	<0.001	907.64	<0.001
Y.B	1	20.06	<0.001	4.47	0.035	36.46	<0.001	65.32	<0.001
Y.C	10	783.81	<0.001	244.58	<0.001	1118.52	<0.001	680.55	<0.001
B.C	7	45.55	<0.001	70.29	<0.001	61.20	<0.001	123.49	<0.001
C(Allele)	10	107.41	<0.001	105.68	<0.001	90.87	<0.001	101.24	<0.001
Y.B.C	7	1.58	0.904	3.11	0.683	4.00	0.549	8.74	0.121
Y.C(Allele)	10	11.12	0.435	7.84	0.727	15.80	0.152	8.64	0.654
B.C(Allele)	7	2.55	0.862	4.48	0.612	3.22	0.781	4.61	0.595
Y.B.C(Allele)	7	7.34	0.292	4.23	0.646	9.73	0.138	6.89	0.332
	Chromosome	GRA (mm^2^)		GRL (mm)		GRW (mm)		L/W	
		Allele		Allele		Allele		Allele	
		Av.	Cd.	Av.	Cd.	Av.	Cd.	Av.	Cd.
	1B	22.20	22.31	7.01	7.04	3.96	3.97	1.77	1.77
	1D	22.39	22.57	7.05	7.08	3.98	3.99	1.77	1.78
	2A	**22.38**	**22.63**	7.08	7.11	3.95	3.98	1.79	1.79
	2D	21.31	21.16	6.95	6.90	3.80	3.81	1.83	1.81
	3A	21.15	20.92	**9.96**	**6.85**	3.77	3.80	**1.85**	**1.81**
	3B	22.54	22.67	7.05	7.05	4.00	4.03	1.77	1.75
	5A	**22.11**	**23.08**	**7.02**	**7.17**	**3.94**	**4.03**	1.78	1.78
	6A	**22.57**	**21.89**	**7.03**	**6.97**	**4.02**	**3.93**	**1.75**	**1.77**
	6B	22.21	22.13	7.02	7.02	3.96	3.95	1.77	1.78
	7B	22.01	22.13	6.91	6.89	3.99	4.01	1.73	1.72
	7D	22.23	22.09	7.05	7.02	3.93	3.93	1.79	1.79
	Average s.e.d	0.123		0.021		0.015		0.006	

Analysis performed for all the agronomic traits studied comprising 553 NILs carrying the Avalon or Cadenza alleles in the introgressed region in two trials (the significant levels are based on the Wald test). (HD = Heading date, PH = Plant height, GRYLD = Grain yield, TGRWT = Thousand grain weight, GRpsqm = Grains per square meter, GRA = Grain area, GRL = Grain length, GRW = Grain width and L/W = Grain shape)

Bold text indicates that the difference between the means of the NILs carrying the Avalon and Cadenza allele is greater than twice the standard error of the difference

### Plant height

For PH, the selected target regions for the development of NILs were on chromosomes 2A, 2D, 3A, 3B, 6A and 6B. Significant differences between Avalon and Cadenza alleles were found for the QTLs on chromosomes 2D, 3A, 3B, 6A and 7D (Tables 5 and 6). For the 3B and 6A QTLs, NILs carrying the Avalon allele were taller than NILs carrying the Cadenza allele. However, on 2D, 3A and 7D QTLs, NILs carrying the Avalon allele were shorter. In all cases, the results agree with previous QTL analysis [36, 37]. Compensatory effects were found between PH and GRYLD such as for the QTLs 3B and 6B in which the reduction of PH was not associated with GRYLD penalty.

The complexity of PH can be reduced by studying its components including EL, PL and internode lengths (1stITL, 2ndITL, 3rdITL, 4thITL and 5thITL) which were measured in 2013 only. For those QTLs that showed significant differences for PH (2D, 3A, 3B, 6A and 7D), the ratio between each PH component and the total PH was analysed. PL had the strongest influence for PH on 3B (1.27 cm), 6A (0.72 cm) and 7D (2.04 cm). On 2D, 1stITL showed the strongest effect (0.72 cm). On 3A, 3rdITL had the strongest influence on PH (1.18 cm). Additional file 2 supporting these results is included.

### Heading date

For HD, the chromosomes specifically selected for NIL development were 1B, 1D, however QTLs for HD were also captured on 3A, 6A and 6B as these were located in regions that also influenced PH and GRYLD. Significant differences were observed for 1B, 1D, 3A, 6A and 6B QTLs (Tables 5 and 6). NILs carrying the Avalon allele flowered later at the 1D QTL whereas those on 1B, 3A, 6A and 6B QTLs flowered earlier. These results are consistent with previous studies [37, 38]. The 1D QTL allow the manipulation of heading date for the purposes of better crop adaptation, with minimal ramifications for GRYLD.

### Grain yield and yield components

For GRYLD, the chromosomes selected for the development of NILs were 2D, 3A, 3B, 5A, 7B and 7D. Significant differences between Avalon and Cadenza alleles were observed for the 1D, 3A and 7B QTLs (Tables 5 and 6). NILs carrying the Cadenza allele in the introgressed region on chromosomes 7B had reduced GRYLD. However, the Cadenza allele on the 1B and 3A QTLs conferred the increased GRYLD.

GRYLD was broken down into its components in order to elucidate which explained the significant differences detected for GRYLD and to identify instances in which variation in components is not translated into variation for GRYLD. For GRpsqm, significant differences were found for the 1D, 3A, 6A, 6B and 7B QTLs (Tables 5 and 6). On 1D and 7B QTLs, NILs carrying the Avalon allele had more grains/m^2^ whereas those on 3A, 6A and 6B QTLs had less grains/m^2^. GRpsqm is the result of the spikes/m^2^ (S), spikelet/spike (s/S) and grains/spikelet (G/S) which were only measured in 2013. For S, the Avalon allele significantly increased the spikes/m^2^ on the 7B QTL. For s/S, significant differences were detected on the 3A, 6A and 7B QTLs. NILs carrying the Cadenza allele had more spikelets/spike. For G/S, significant differences between Avalon and Cadenza alleles were observed for the 2A, 3A and 6A QTLs. These had effects in opposite directions; NILs carrying Cadenza allele on 3A and 6A had more grains per spikelet whereas those on 2A had less grains. An additional file supporting these results is included (see Additional file 3).

For TGRWT, significant differences were found for the 2A, 5A and 6A QTLs (Tables 5 and 6). The Avalon allele increased grain weight on 6A, whereas Cadenza provided the increased grain weight on 2A and 5A. TGRWT can be further dissected into individual components including GRL, GRW, GRA and L/W. Three QTLs for GRA (2A, 5A and 6A) and GRL (3A, 5A and 6A) and two for GRW (5A and 6A) and L/W ratio (3A and 6A) showed significant differences.

In our study, GRYLD was driven by GRpsqm as the QTLs that showed significant difference between alleles for GRYLD were also significant for GRpsqm. Only the 6A QTL showed significant TGRWT effects linked to GRpsqm. Avalon allele increased TGRWT through an increasing in GRA, GRL and GRW and reduced GRpsqm by reducing spikelet/spike (s/S) and grains/spikelet (G/S).

### Epistatic interactions

The mixed model fitted was used to determine possible epistasis by the analysis of the Background × Chromosome (Allele) interaction (B × C(A)) which indicates possible QTL × background or QTL × donor segment interactions (Tables 5, 6 and 7). HD (*p-value* = 0.004) and PH (*p-value* = 0.050) showed significant (B × C(A)) interaction. For HD, epistatic interactions were detected for the 3A and 6B QTLs. In both cases, the magnitude of the differences was greater in the Avalon background (34 °C and 21 °C on 3A and 6B, respectively) compared to the Cadenza background (16 °C on 3A and 9 °C on 6B). These QTLs also showed epistatic interactions for other traits. On 3A QTL, background effects were found for GRYLD whereas on 6B were detected for PH, an increase of GRYLD and PH was greater in the Cadenza background (3.42 cm on 6B and 0.88 T/Ha on 3A). On 3A, the greater GRYLD effects on the Cadenza background can be explained by the reduction of PH (7.31 and 4.65 cm in the Avalon background and the Cadenza background, respectively) and the increase of Harvest Index (HI) (0.01 in the Avalon background and 0.02 in the Cadenza background) through the decrease in Biomass (3.47 in the Avalon background and 3.10 in the Cadenza background) in 2013. In wheat breeding, HI and GRYLD have been increased by reducing PH providing crop advantage as not only the risk of lodging is reduced also the distribution of resources in the plant is better [49]. For GRYLD, epistatic interactions were also detected on 2A QTL. The magnitude of the differences was greater in the Avalon background (0.53 T/Ha) compared to the Cadenza background (0.03 T/Ha).

**Table 7:** Average values for the two groups of NILs (carrying the Avalon or Cadenza alleles in the QTL region) based on the chromosome and background

		PH (cm)		HD (^o^ C)		GRYLD (T/Ha)	
		Allele		Allele		Allele	
Background	Chromosome	Av.	Cd.	Av.	Cd.	Av.	Cd.
Avalon	1B	80.71	81.3	1572	1588	9.29	9.60
	1D	76.12	74.65	1580	1551	9.30	9.02
	2A	73.94	73.54	1579	1567	8.55	9.08
	2D	76.63	80.75	1566	1564	9.75	9.91
	3A	74.34	70.49	1565	1599	9.25	9.60
	3B	75.34	72.05	1578	1581	-	-
	5A	73.93	75.64	1576	1573	8.92	9.00
	6A	75.51	71.62	1581	1599	9.39	9.20
	6B	75.65	75.47	1574	1595	9.31	9.45
	7B	75.42	73.52	1592	1582	9.84	9.03
	7D	74.11	79.40	1579	1581	9.46	9.30
Cadenza	1B	77.59	78.43	1532	1555	9.56	9.98
	1D	79.31	78.18	1568	1546	9.96	9.64
	2A	81.89	81.16	1556	1548	9.87	9.83
	2D	77.59	82.80	1555	1545	-	-
	3A	75.08	79.44	1545	1561	8.99	9.87
	3B	82.92	77.11	1554	1554	9.73	9.78
	5A	-	-	-	-	-	-
	6A	83.44	77.77	1536	1550	9.26	9.35
	6B	75.59	79.01	1537	1546	9.53	9.81
	7B	-	-	-	-	-	-
	7D	-	-	-	-	-	-
Average s.e.d	1.336	5.461	0.247				

## Discussion

Previous QTL studies using the Avalon × Cadenza DH population identified the location of QTL determining HD, PH and GRYLD [15, 36–38]. The development of NILs using marker-assisted selection helped to validate these QTLs and will define favourable allelic combinations to develop superior genotypes adapted to a target environment. In the present study, a set of NILs were developed using two recurrent parents (Avalon or Cadenza). This reciprocal approach allowed us to confirm not only the effect of the QTLs, but also determine if the expression of these QTLs were specific for the recurrent parent used in the backcrossing scheme or year.

The 553 BC_2_ NILs are divided in two groups depending on the background (Avalon and Cadenza). NILs carrying the Cadenza background were taller, flowered earlier and had increased GRYLD and yield components in both years. Part of these differences can be explained by *Vrn-A1* (on 5A) and *Rht-D1* (on 4D). Although the NILs were not genotyped for *Vrn-A1* and *Rht-D1* we expected them to be segregating as the recurrent parents differed for *Vrn-A1* and *Rht-D1*. Avalon carries the recessive *vrn-A1* and dwarf *Rht-D1b* alleles whereas Cadenza carries the dominant *Vrn-A1a* and the wild type *Rht-D1a* allele. *Rht-D1* had no effect on flowering time [50]. Although plants experienced warmer temperatures during the vegetative phase in 2014, there was still enough vernalizing cold as the difference for HD between NIL groups were similar for both years (27.62 and 26.28° days in 2013 and 2014, respectively). However, warmer temperatures and early sowing allowed NILs carrying the Cadenza background (spring types) to develop more quickly than NILs carrying the Avalon background (winter types), then slowed growth and development due to lower temperatures during winter without affecting plant survival. This could explain the inconsistency in the magnitude of the effects between NIL groups for PH (1.22 cm in 2013 and 4.24 cm in 2014) which can also be associated with higher GRYLD and yield components.

### Comparison with previous studies

The present study validated the HD, PH and GRYLD QTLs identified by [15, 36–38]. However, some differences were observed with the direction of the QTL effect in the NILs when compared to those expected from the original A × C DH population. These differences are probably due to G × E interactions. For HD, the Avalon allele delayed flowering for the 7B QTL whereas in the original DH population the delay was detected for the Cadenza allele. For the 6A QTL in the NILs, the Cadenza allele increased the grain length whereas the Cadenza allele decreased it in the original population. In addition, this study allowed the identification of QTLs that showed significant differences which were not detected in the original DH population such as 2A for HD, 7D for PH, 1B and 7B for GRYLD.

An additional point to consider is the magnitude of the effects, so the effect in the ‘new’ background is the same as the effect estimated in the original DH population. An additional file shows the comparison between the additive effects of the Avalon × Cadenza DH population and the NILs for PH and HD (see Additional file 4). Similar QTL effects were observed in most of the cases, with the exception of the 2D QTL for PH and 1D and 3A QTLs HD. These discrepancies can be explained by QTL × E interaction, QTL× background interaction and weak phenotypic effects [51, 52]. These results agree with [37] who detected QTL × Env interactions on 1D and 3A for HD. Evidence of epistatic interactions were found in the original DH population (unpublished data) which can also explain these discrepancies. Further genotyping of the NILs should provide more evidence to clarify these interactions, observed as background effects in this study.

### Common effects among traits

In our study, GRYLD was driven by GRpsqm as all the QTLs that showed significant difference between alleles for GRYLD were also significant for GRpsqm (1B, 3A and 7B). This was consistent with the highly positive correlation coefficient between both traits (0.80 in 2013 and 0.89 in 2014). Same effect direction was observed for GRYLD and GRpsqm. For GRYLD, differences were almost significant (dropping below significant threshold) on 1D QTL. It was interesting that the increasing allele on 1B and 3A was Cadenza whereas on 1D and 7B the increasing allele was Avalon. For the 1B QTL, the Avalon allele reduced GRYLD and HD with neutral effects on PH. The 1D QTL was also associated with HD suggesting that the early allele (Cadenza) would increase GRYLD with neutral effects on PH. These results are consistent with the effects detected in the Avalon × Cadenza DH population [37]. For the 3A QTL, GRYLD difference was associated with HD and PH; the late allele (Cadenza) conferred tallness, GRYLD and GRpsqm increased with neutral effects on TGRWT. The combination of the Cadenza allele on 1D and 3A will result in an increase of GRYLD with neutral effects on HD. It has been shown [38] the segregation of both HD QTLs could confer a crop performance advantages. The direction of the effects for all traits agreed with the results obtained by Griffiths et al. [36, 38] and Ma et al. [37], suggesting possible tight linkage or pleiotropic effects. In addition, the Cadenza allele increased the roundness of the grain by decreasing grain length and the L/W ratio. QTLs for GRL and the L/W ratio in the Avalon × Cadenza DH population were found by Gegas et al. [15]; the Avalon allele increased grain length and the L/W ratio. The increased GRpsqm from the Cadenza allele can be explained by the increase in the grains/spikelet and spikelet/spike. On chromosome 3A, clusters of QTLs for GRYLD, yield components, PH and HD have been found in previous studies [24, 53–56]. It is not surprising that our results agreed with the previous findings as the interval defined by the SSR markers used for the NILs development on 3A is 48 cM based on the Avalon ×  Cadenza reference map (http://www.cerealsdb.uk.net). Ma et al. [37] found two closely linked GRYLD QTLs, *qGY-psr-3A.1* and *qGY-psr-3A.2* at around 73 cM and 87 cM, respectively. The *qGY-psr-3A.1* co-located with PH (*qPH-psr-3A*) and HD (*qEM-psr-3A*) QTLs and the *qGY-psr-3A.2* with GRpsqm (*qGN-psr-3A*) and GRL (*qGRL-psr-3A*) QTLs with the Cadenza alleles increasing all the traits except GRL. Therefore, selecting for the Avalon allele for the *qGY-psr-3A.1* and for the Cadenza allele for the *qGY-psr-3A.2* should provide an elite line with early flowering, shorter stature and increased in GRYLD and GRpsqm with round grains. However, high-resolution mapping together with the development of recombinants will be required to break this linkage between these QTLs or confirm pleiotropic effects. For the 7B QTL, the Avalon allele increased GRYLD through an increased in GRpsqm with neutral effects on PH and HD.

In our study, no common QTLs were found between GRYLD and TGRWT. Only the 6A QTL showed significant TGRWT effects linked to GRpsqm. Our data suggested that TGRWT is mainly explained by GRA and GRW on the 6A QTL. The early allele (Avalon) associated with TGWRT via increasing GRA, GRL and GRW and reduced GRpsqm by reducing spikelet/spike (s/S) and grains/spikelet (G/S) with some height penalty. The SSR markers (barc23 to gwm570) used for the foreground selection on 6A include the TGWRT QTL reported by Simmonds et al. [2] in the Spark × Rialto population; however, in this study it was expressed as a GRYLD QTL. Griffiths et al. [36] detected common QTLs for PH and HD on 6A with effects in opposite directions which support our results. For the 5A QTL, the Cadenza allele increased TGWRT by increasing GRA, GRL and GRW with neutral effects for PH and HD penalty. In the Avalon × Cadenza DH population, QTLs controlling TGRWT, GRpsqm, GRW, GRL, PH and HD were detected on chromosome 5A [15].

In the Avalon background, the introgression of the five target regions on chromosomes 1D, 3A, 5A, 6A and 7B should provide an elite line with increased GRYLD (1.87 T/Ha), TGRWT (8.56 g), Grpsqm (5364 grains/m^2^), GRA (2.49 mm^2^), GRL (0.36 mm), PH (14.73 cm) and HD (94° days to heading). In the Cadenza background, the introgression of 3A and 6A target regions will increase GRYLD (1.30 T/Ha), TGRWT (3.43 g), Grpsqm (2830 grains/m^2^), GRA (1.04 mm^2^), GRL (0.21 mm), PH (9.99 cm) and HD (30° days to heading). An alternative approach to take advantage of QTL information was proposed by Ma et al. (2015). They used a ‘breeding by design’ simulated approach to define the best two parental DH lines and selection methods in order to develop a target genotype that performed well to UK conditions. These crosses are now being conducted and will verify the simulation results.

## Conclusion

In the present study we validate eleven QTLs determining PH, HD and GRYLD previously identified in the UK reference DH population Avalon × Cadenza in two different backgrounds. The development of 553 NILs provides a unique opportunity to assess the ‘real’ QTL effects and epistatic interactions estimated by looking at the background effects which will allow breeders to define favourable allelic combinations to develop superior genotypes adapted to a target environment. In addition, these results provides an important first step to further fine map of these traits.

## Abbrevations

DH, doubled haploid; EL, ear length; Eps, earliness per se; G/S, grains/spikelet; GRA, grain area; GRL, grain length; GRpsqm, grain number; GRW, grain width; GRYLD, grain yield; HD, heading date; HI, harvest index; L/W, grain shape; MAS, marker-assisted selection; NILs, near isogenic lines; PCA, principal component analysis; PH, crop height; PL, peduncle; Ppd, photoperiod; QTLs, quantitative trait loci; s/S, spikelet/spike; S, spikes/m2; SSR, microsatellite; TGRWT, thousand grain weight; Vrn, vernalization; WGIN, Wheat Genetic Improvement Network

## Additional files

Additional file 1: Figure S1. Means of minimum (blue line) and maximum (red line) temperatures and cumulated rainfall (blue bars) during the months of the growth cycle for both trials. (PDF 95 kb) Additional file 2: Figure S2. Difference between Avalon and Cadenza alleles for the ratio between the lenghts of each PH component and the total PH for the chromosome that showed significant differences for plant height (values above zero indicates that the Avalon allele increased height whereas below zero the Cadenza allele reduced height; ear length (EL), peduncle (PL) and internode lengths (from the first to the fifth counted from the top, abbreviated as 1stITL, 2ndITL, 3rdITL, 4thITL, 5thITL); * *P*-value < 0.05; ** *P*-value <0.01 and *** *P*-value < 0.001). (PDF 165 kb) Additional file 3: Table S3. (a) Summary of the mixed model analysis performed for the grain number components studied comprising 553 NILs carrying the Avalon or Cadenza alleles in the introgressed region in 2013. (b) Average values for the two groups (carrying the Avalon or Cadenza alleles in the QTL region) based on the chromosome and background in 2013. Significant difference between Avalon and Cadenza alleles are highlighted in bold (spikes/m2 (S), spikelet/spike (s/S) and grains/spikelet (G/S). (PDF 111 kb) Additional file 4: Figure S3. Comparions between the magnitude of the effects of the A × C DH population (red) and the NILs for heading date (a) and plant heigth (b) (2013 and 2014 are indicated in bakc and grey color, respectively; the Avalon background is showed in dashed line whereas the Cadenza background is showed in straigth line). (PDF 91 kb)
