# Supplementary figures and images for: Application of a library of near isogenic lines to understand context dependent expression of QTL for grain yield and adaptive traits in bread wheat
Source: BMC Plant Biol. 2016 Jul 19;16:161. doi: 10.1186/s12870-016-0849-6 (PMC4952066; doi:10.1186/s12870-016-0849-6)

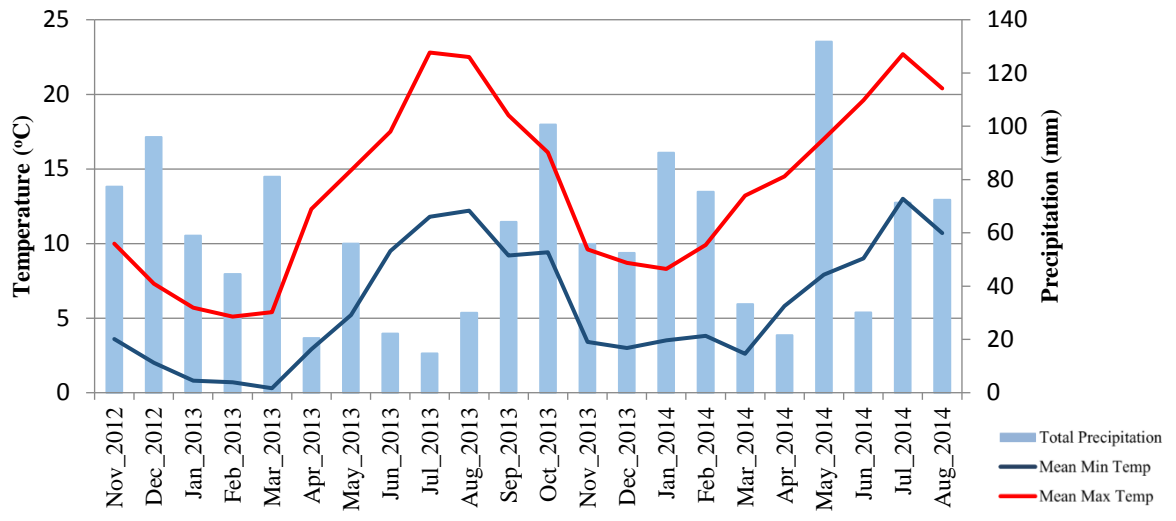

Supplement: Additional file 1: Figure S1. — Means of minimum (blue line) and maximum (red line) temperatures and cumulated rainfall (blue bars) during the months of the growth cycle for both trials. (PDF 95 kb) [file 12870_2016_849_MOESM1_ESM.pdf]

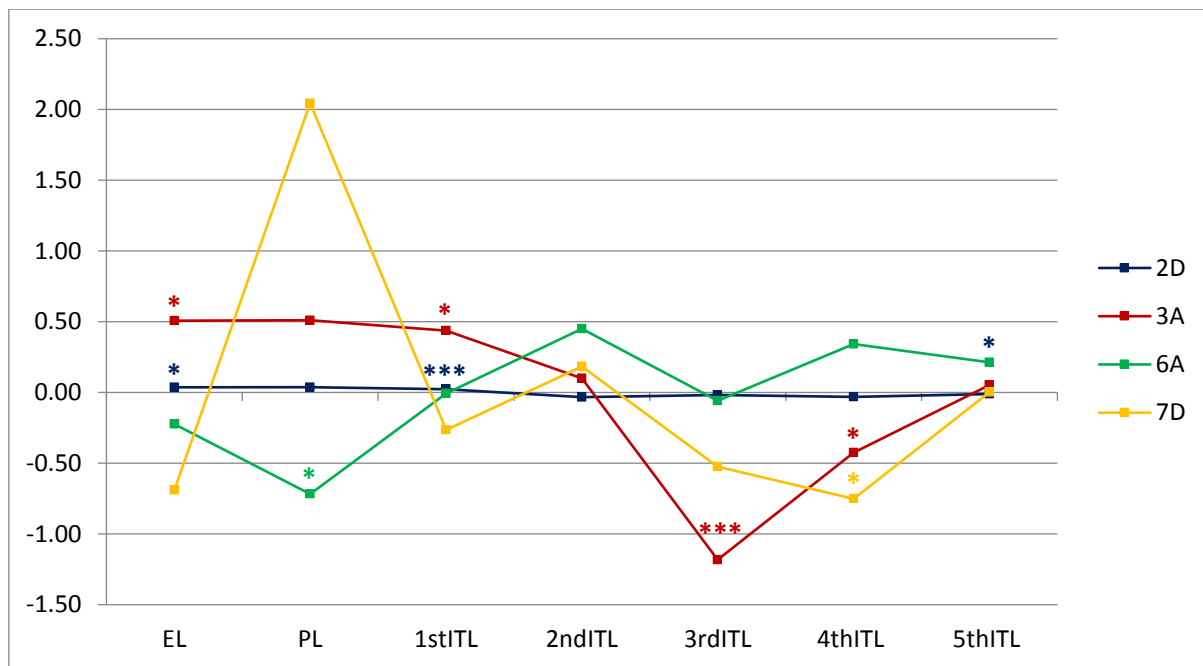

Supplement: Additional file 2: Figure S2. — Difference between Avalon and Cadenza alleles for the ratio between the lenghts of each PH component and the total PH for the chromosome that showed significant differences for plant height (values above zero indicates that the Avalon allele increased height whereas below zero the Cadenza allele reduced height; ear length (EL), peduncle (PL) and internode lengths (from the first to the fifth counted from the top, abbreviated as 1stITL, 2ndITL, 3rdITL, 4thITL, 5thITL); * P-value < 0.05; ** P-value <0.01 and *** P-value < 0.001). (PDF 165 kb) [file 12870_2016_849_MOESM2_ESM.pdf]

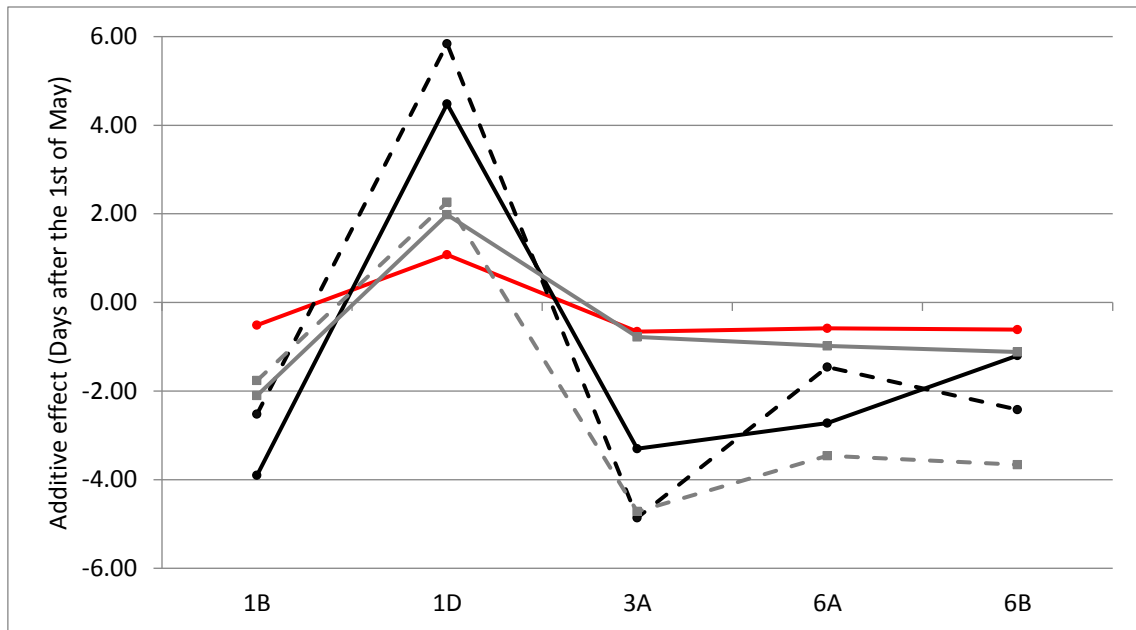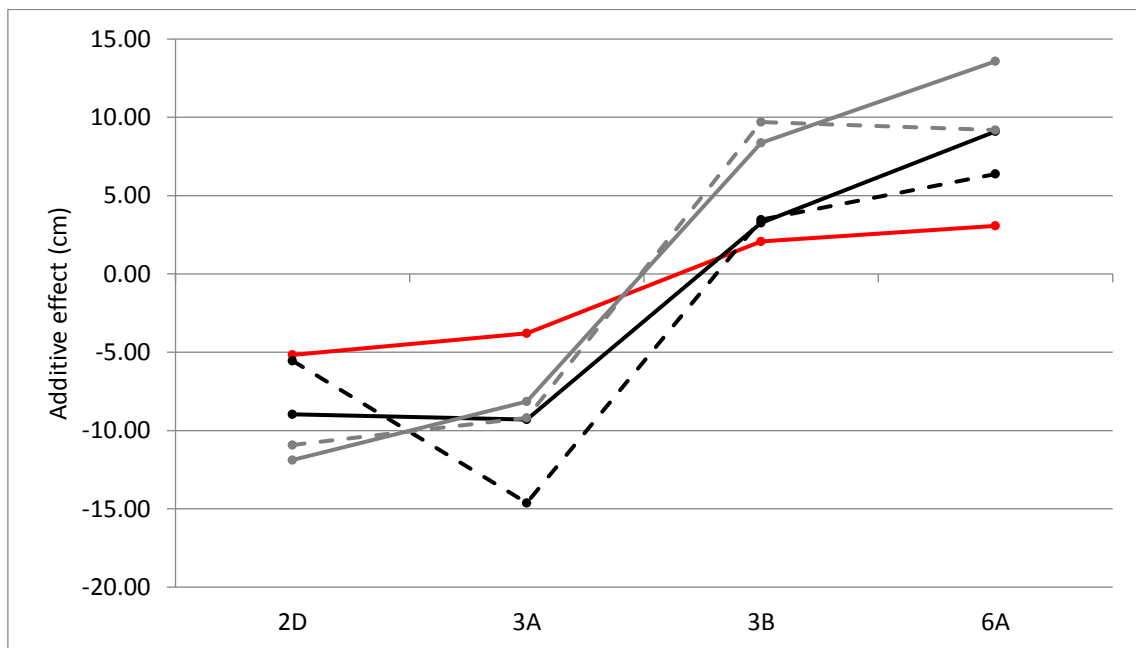

Supplement: Additional file 4: Figure S3. — Comparions between the magnitude of the effects of the A × C DH population (red) and the NILs for heading date (a) and plant heigth (b) (2013 and 2014 are indicated in bakc and grey color, respectively; the Avalon background is showed in dashed line whereas the Cadenza background is showed in straigth line). (PDF 91 kb) [file 12870_2016_849_MOESM4_ESM.pdf]
