# Supplementary material for: Application of a library of near isogenic lines to understand context dependent expression of QTL for grain yield and adaptive traits in bread wheat
Source: BMC Plant Biol. 2016 Jul 19;16:161. doi: 10.1186/s12870-016-0849-6 (PMC4952066; doi:10.1186/s12870-016-0849-6)
Supplement: Additional file 3: Table S3. — (a) Summary of the mixed model analysis performed for the grain number components studied comprising 553 NILs carrying the Avalon or Cadenza alleles in the introgressed region in 2013. (b) Average values for the two groups (carrying the Avalon or Cadenza alleles in the QTL region) based on the chromosome and background in 2013. Significant difference between Avalon and Cadenza alleles are highlighted in bold (spikes/m2 (S), spikelet/spike (s/S) and grains/spikelet (G/S). (PDF 111 kb) [file 12870_2016_849_MOESM3_ESM.pdf]

(a)

Source of variation	d.f.	S		s/S		G/S	
		Wald-test	p-value	Wald-test	p-value	Wald-test	p-value
Background (B)	1	3.44	0.064	167.84	<0.001	43.77	<0.001
Chromosome (C)	10	14.32	0.164	41.37	<0.001	41.83	<0.001
B.C	7	24.47	<0.001	27.75	<0.001	14.93	0.012
C(Allele)	10	5.99	0.873	70.60	<0.001	30.51	0.002
B.C(Allele)	7	3.83	0.700	11.71	0.072	5.40	0.495

(b)

	S		s/S		G/S	
	Allele		Allele		Allele	
Chromosome	Avalon	Cadenza	Avalon	Cadenza	Avalon	Cadenza
1B	350.80	353.20	19.57	19.47	2.59	2.54
1D	350.50	344.90	19.65	19.44	2.53	2.55
2A	326.00	330.20	19.57	19.71	**2.70**	**2.47**
2D	328.00	332.30	19.14	19.17	2.75	2.81
3A	339.40	338.10	**19.33**	**20.16**	**2.53**	**2.67**
3B	346.90	350.40	20.17	20.03	2.51	2.52
5A	342.90	352.30	19.07	18.52	2.53	2.38
6A	344.10	343.00	**19.12**	**19.70**	**2.48**	**2.54**
6B	345.20	345.90	19.54	19.77	2.61	2.63
7B	**357.30**	**303.70**	**18.76**	**19.41**	2.61	2.61
7D	351.90	341.80	19.35	19.55	2.66	2.62
Average s.e.d	10.63		0.182		0.057	

	S		s/S		G/S	
	Allele		Allele		Allele	
Chromosome	Avalon	Cadenza	Avalon	Cadenza	Avalon	Cadenza
1B	350.80	353.20	19.57	19.47	2.59	2.54
1D	350.50	344.90	19.65	19.44	2.53	2.55
2A	326.00	330.20	19.57	19.71	**2.70**	**2.47**
2D	328.00	332.30	19.14	19.17	2.75	2.81
3A	339.40	338.10	**19.33**	**20.16**	**2.53**	**2.67**
3B	346.90	350.40	20.17	20.03	2.51	2.52
5A	342.90	352.30	19.07	18.52	2.53	2.38
6A	344.10	343.00	**19.12**	**19.70**	**2.48**	**2.54**
6B	345.20	345.90	19.54	19.77	2.61	2.63
7B	**357.30**	**303.70**	**18.76**	**19.41**	2.61	2.61
7D	351.90	341.80	19.35	19.55	2.66	2.62
Average s.e.d	10.63		0.182		0.057	
